# Oesophageal foreign bodies in cats: Clinical and anatomic findings

**DOI:** 10.1371/journal.pone.0233983

**Published:** 2020-06-02

**Authors:** Naglaa A. Abd Elkader, Ibrahim A. Emam, Haithem A. Farghali, Daghash S. M., Noha Y. Salem

**Affiliations:** 1 Surgery, Anesthesiology and Radiology Department, Faculty of Veterinary Medicine, Cairo University, Giza, Egypt; 2 Anatomy and Embryology Department, Faculty of Veterinary Medicine, Cairo University, Giza, Egypt; 3 Internal Medicine and Infectious Diseases Department, Faculty of Veterinary Medicine, Cairo University, Giza, Egypt; University of Lincoln, UNITED KINGDOM

## Abstract

**Background:**

Anatomical feline models can aid in understanding the relationships between clinical findings and anatomical features and the course of foreign bodies passing through the oesophagus. This study has two goals 1) to assess feline oesophageal foreign bodies in feline patients using physical, radiologic and endoscopic examination and, how their location influences treatment plans and complications. 2) How the anatomical sharp angle of the oesophagus contribute to foreign body lodgement. Thirty-five cats were enrolled in this study; 30 of them were clinically ill, and five cats were used for anatomical study.

**Results:**

Cats with clinical signs underwent complete clinical and radiologic examination. Endoscopy was performed in only five cases. The site with the highest occurrence of foreign body lodgement was the oesophageal entrance, caudal to the pharynx (63.3%), followed by the thoracic inlet (26.7%) and the mid-cervical region of the oesophagus (10%). Two types of foreign bodies were identified: sewing needles (25/30) and bone (5/30). Radiography was able to identify the location and nature of the foreign body in all 30 affected cats. Therapeutic regimens were applied according to the nature and location of the foreign body and any associated complications. Removal of the foreign body was achieved using Rochester pean artery forceps in 17/30 cases, using full surgical intervention in 8/30 cases, and during endoscopy in 5/30 cases.

**Conclusion:**

The results suggest that the location of the foreign body is strongly related to combination of consumed foreign body type and anatomic features of the cat oesophagus. The feline oesophagus has a variety of sharp angles that facilitate the entrapment of rigid linear and angular foreign bodies. Radiographic imaging remains the most frequently used diagnostic modality for determining the lodgement site and nature of radiopaque foreign bodies. Over all complication rate was low (6/30).

## Introduction

In domestic animals, the oesophagus is described as a tube that connects the pharynx to the stomach [1, 2].

Cats like to play with toys and small items, which makes them susceptible to the lodging of foreign bodies in the oral cavity or oesophagus. Oesophageal foreign bodies are not common but are problematic. A wide range of foreign bodies has been identified; needles, fish bones and other bones are those most frequently encountered [3].

Though the clinical presentation of oral or oesophageal foreign body lodgement depends mainly on the lodgement site—the object's size, the length of time of partial or complete oesophageal obstruction could also influence signs, the common classical presentation of oesophageal foreign body lodgement is regurgitation, dysphagia, discomfort and respiratory distress [4]. If the foreign body is not addressed via medical intervention, serious consequences may ensue [5] as pleural inflammation, oesophagitis, aspiration pneumonia and mortality are among the possible complications [6].

Diagnostic procedures depend predominantly on history combined with the clinical findings. Swallowing a foreign body followed by vomiting elicits an abdominal work-up while swallowing a foreign body followed regurgitation and retching requires oral examination and oesophageal imaging, which may be indicative of foreign body involvement [7]. Plain and contrast radiography play important roles in confirming the diagnosis [8]. Radiography provides information about not only the site of lodgement but also possible complications [3]. Endoscopic examination of the oesophagus is among the most useful diagnostic tools for oesophageal disease assessment [5].

The formulation of a treatment plan is premised on factors such as the size, site and nature of the foreign object [4]. However, endoscopic removal of foreign bodies has been advocated and demonstrated in previous studies [4,5,9].

The lodgement of a foreign body in the cat oesophagus is associated with the anatomical features of the oesophagus, especially its angulations and bending course. However, the nature of the foreign body might also play a role [10]. Consideration of the anatomy of the oesophagus may provide insight into clinical findings and increase our understanding of the movement of foreign bodies through the oesophagus.

This study has two goals 1) to review cases of oesophageal foreign bodies in feline patients using physical, radiologic and endoscopic examination, treatment plans and complications, 2) to investigate the relation between the anatomical sharp angles of the oesophagus, location of entrapped foreign bodies and, how anatomical curvatures contribute to foreign body obstruction in cats.

## Material and methods

Ethical approval (CU-ӀӀ-F-17-18) for this study was provided by Cairo University Institutional Animal Care and Use Committee (CU- IACUC) Veterinary Medical and Agricultural Sciences Sector.

### Study animals

Thirty cats of different breeds and ages admitted to the small animal surgery clinic of the Faculty of Veterinary Medicine, Cairo University, were used in the current study. Information on clinical signs and the cats' medical history was collected at admission, and each owner was asked if he/she witnessed ingestion of the foreign body and the time that had elapsed between foreign body ingestion and admission. Owner consents were given verbally from all participants. Selection criteria for patients include owner witnessing swallowing of foreign body, clinical signs and identifying radiopaque object by radiographic imaging. The affected cats in this study were divided based on history, owner observations and definitive identification of a foreign body in the oesophagus by radiographic imaging of radiopaque object. An additional five apparently healthy cats were used for anatomical study.

### Clinical examination

Physical examination of the oesophagus–includes palpation of cervical oesophagus to detect mass, distension or foreign body- was performed, and clinical signs of the affected cats were recorded. The location of foreign body was determined based on the results of physical, radiologic and endoscopic examinations and cats were divided into three groups based on these findings. The nature of each foreign body was also determined based on the above-described methods. Data on each cat are presented in Table 1.

**Table 1:** Data from thirty cats with oesophageal foreign bodies.

Case no.	Age (Y)	Sex	Breed	Foreign body location	Foreign body type
1	≤1	Male	Mixed breed	Oesophageal entrance caudal to pharynx	Sewing needle
2	1 to ≤ 2	Female	Persian (Shiraz)	Oesophageal entrance caudal to pharynx	Needle
3	≤1	Male	Persian	Oesophageal entrance caudal to pharynx	Sewing needle
4	1 to ≤ 2	Male	Persian	Oesophageal entrance caudal to pharynx	Sewing needle
5	1 to ≤ 2	Male	Mixed breed	Oesophageal entrance caudal to pharynx	Sewing needle
6	1 to ≤ 2	Male	Mixed breed	Oesophageal entrance caudal to pharynx	Sewing needle
7	≤1	Female	Persian	Oesophageal entrance caudal to pharynx	Sewing needle
8	≤1	Female	Mixed breed	Oesophageal entrance caudal to pharynx	Bone
9	1 to ≤ 2	Male	Persian	Oesophageal entrance caudal to pharynx	Sewing needle
10	1 to ≤ 2	Male	Persian	Oesophageal entrance caudal to pharynx	Sewing needle
11	1 to ≤ 2	Female	Mixed breed	Oesophageal entrance caudal to pharynx	Bone
12	1 to ≤ 2	Male	Persian	Oesophageal entrance caudal to pharynx	Sewing needle
13	≤1	Female	Mixed breed	Oesophageal entrance caudal to pharynx	Sewing needle
14	1 to ≤ 2	Male	Mixed breed	Oesophageal entrance caudal to pharynx	Sewing needle
15	≤1	Female	Persian	Oesophageal entrance caudal to pharynx	Sewing needle
16	1 to ≤ 2	Male	Mixed breed	Oesophageal entrance caudal to pharynx	Sewing needle
17	≤1	Male	Persian	Oesophageal entrance caudal to pharynx	Sewing needle
18	≤1	Female	Persian	Oesophageal entrance caudal to pharynx	Sewing needle
19	1 to ≤ 2	Male	Persian	Oesophageal entrance caudal to pharynx	Sewing needle
20	1 to ≤ 2	Male	Mixed breed	Mid-cervical region of oesophagus	Bone
21	≤1 Y	Female	Mixed breed	Mid-cervical region of oesophagus	Bone
22	1 to ≤ 2	Female	Persian	Mid-cervical region of oesophagus	Bone
23	1 to ≤ 2	Female	Mixed breed	Thoracic inlet	Needle
24	1 to ≤ 2	Female	Persian	Thoracic inlet	Sewing needle
25	1 to ≤ 2	Male	Persian	Thoracic inlet	Sewing needle
26	≤1	Female	Persian	Thoracic inlet	Sewing needle
27	≤1	Male	Mixed breed	Thoracic inlet	Sewing needle
28	1 to ≤ 2	Female	Persian	Thoracic inlet	Sewing needle
29	≤1	Female	Mixed breed	Thoracic inlet	Sewing needle
30	1-≤ 2	Female	Mixed breed	Thoracic inlet	Sewing needle

### Diagnostic modalities

#### Radiology

Radiographic imaging was performed to identify the location of the foreign body using a Fischer X-ray unit. The radiographic parameters were 40–44 kV, 100 mAs at 0.1 second and 100 FFD for the lateral view and 42–46 kV, 100 mAs at 0.1 second and 100 FFD for the ventro-dorsal view for all cats.

#### Endoscopic examination

Endoscopic examination was performed in some cats under general anaesthesia as part of the treatment plans. Atropine sulphate (1%at 0.05–0.1 mg/kg b.wt.; Adwia Co., S.A.E, Egypt) and xylazine (Xyla-Ject® 2% at 1 mg/kg b.wt.; Adwia Co., S.A.E, Egypt) were used as pre-medication, followed by Ketamine at 10–20 mg/kg b.wt. (Sigma-Tec, Egypt) for induction and maintenance [11]. Cats were intubated by endotracheal tube. Then, endoscopy (oesophageoscopy) was performed according to previously published methods [12].

#### Treatment plans and complications

After identifying the type and location of the foreign body—Six cats were admitted with complications and were addressed according to its nature- a treatment plan was formulated. There were three treatment options: 1) foreign body removal during endoscopy, 2) removal of the foreign body using Rochester pean artery forceps, and 3) surgical removal. Briefly, surgical intervention was done under complete aseptic condition and general anaesthesia. The following protocol was followed: Skin- incision only for removing the subcutaneous sharp foreign body that penetrates oesophageal wall forming subcutaneous reaction. Skin and muscles incision of affected area was made for foreign body retrieval. After removal, muscles, subcutaneous tissues were sutured using simple continuous suture pattern and skin by simple interrupted suture pattern via absorbable suture material (Vicryl 3–0). Cats were discharged and owner instructed to daily dressing using skin antiseptic solution (Betadine^®^ BID for 7–10 days) and amoxicillin-clavulante 12.5mg/kg BID/5-7 days.

#### Anatomical considerations

To help in understanding the movement of foreign bodies and the anatomical predispositions for foreign body lodgement in certain areas of oesophagus, an anatomical model was created.

Five adult, clinically healthy cats (1–2 years) of both sexes were used to create the model. Cats were obtained from Anatomy Department, faculty of veterinary medicine -Cairo University which had the right to have cadavers of multiple species for educational purposes for both under and post graduate students. Each cat was sedated using xylazine HCl (1 mg/kg). Then, negative contrast oesophagography was performed using atmospheric air to inflate the oesophagus via catheter. Next, barium oesophagography was performed using a Nelaton catheter at 52 kV and 14 mAs using a Poskom digital X-ray unit model (No. PXP-40HF, Goyang, Korea).

The cats were sedated using xylazine HCl (1 mg/kg) and then humanely euthanized using intravenous administration of thiopentone sodium, dose to effect. Death was confirmed by the absence of chest movement, heart rate, and pulse. Three of the euthanized cats were fixed in formalin solution (10% formalin, 4% phenol, 1% glycerin) and left for 5 days in a cold room [13] before manual dissection. The two remaining euthanized cats were used for corrosion casting; the oesophagus was intubated with a Nelaton catheter and filled with 120 mL of green-coloured Kemapoxy 150 (CMB, Egypt). Specimens were left for three days to solidify before being macerated using KOH 20% [14]. Nomenclature was adopted according to Nomina Anatomica Veterinaria [15].

## Results

### Signs and physical findings

All cats admitted with history of off-food complain and varying degree of other signs. Anorexia, lethargy, and dysphagia were the most frequent recorded complaints. Pain in the cervical region was detected via physical examination. Persian (Shiraz) cats were diagnosed with oesophageal foreign body more frequently than mixed breed cats (16/30, 53.3%; 14/30, 46.7% respectively). In addition, cats aged 1–2 years appeared to have a higher affinity for ingesting foreign bodies (18/30, 60%) than did cats aged less than 1 year (12/30, 40%). Male and female cats were approximately equally affected (Table 1). The owner witnessed the ingestion of the foreign body in 22 out of 30 (73.33%) cases.

### Diagnostic aids

X-ray imaging was performed for all cats; radiography successfully identified the location and possible type of foreign body involved in 30/30 of the clinical cases. Endoscopic examination was performed in 5/30 cases to help establish a definite diagnosis and to extract the foreign body.

### Characterization of the nature and location of foreign bodies

Two types of foreign body were identified in this study, as shown in Table 1. Sewing needles were the most common body identified (25/30, 83.3%), followed by bones (chicken and fish bones; 5/30, 16.7%).

The locations and associated complications of the foreign bodies are presented in Table 2.

**Table 2:** Locations, findings and therapeutic plans associated with oesophageal foreign bodies in cats.

Location	Total number/ percentage	Findings	Therapeutic plan
**At oesophageal entrance caudal to pharynx**	19 (63.3%)	Thirteen cats with sewing needle protruding from oral cavity.	Retrieval of foreign body using artery forceps
		One cat with needle protruding from epiglottis.	
		One cat with needle protruding from oesophageal wall.	
		Two cats with oesophageal abscess formation.	
		Two cats with bone foreign body.	Retrieval of foreign body by oesophagoscopy
**Mid-cervical region of oesophagus**	3 (10%)	Bones were identified in all three cats.	
**Terminal region of oesophagus at thoracic inlet**	8 (26.7)	Six cats with needle without complications.	Retrieval of foreign body by surgical intervention
		Two cats with needle perforation at the level of the 6th to 7th cervical vertebrae; soft tissue reaction was observed in pectoral muscles.	

**Fig 1:**
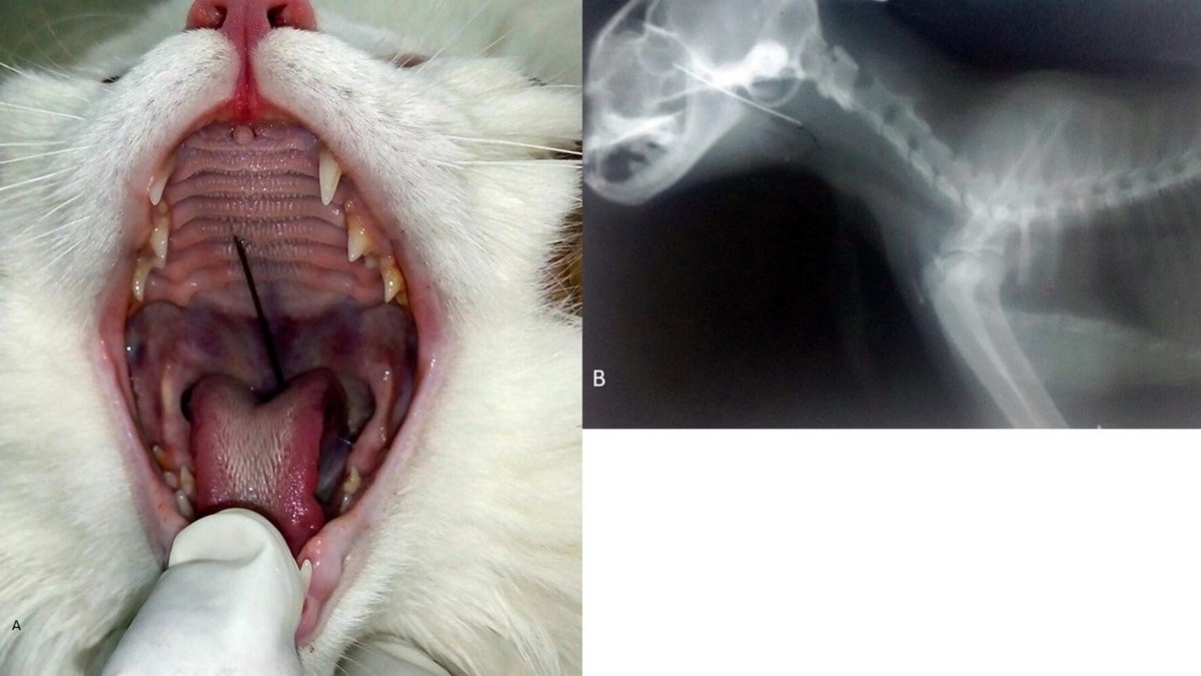
Clinical and radiographic findings regarding oesophageal foreign bodies at the oesophageal entrance (cervical region). a. Clinical photograph of mixed breed cat showing protruded sewing needle in oral cavity. b. Lateral radiographic view of cat showing radiopaque needle.

**Fig 2:**
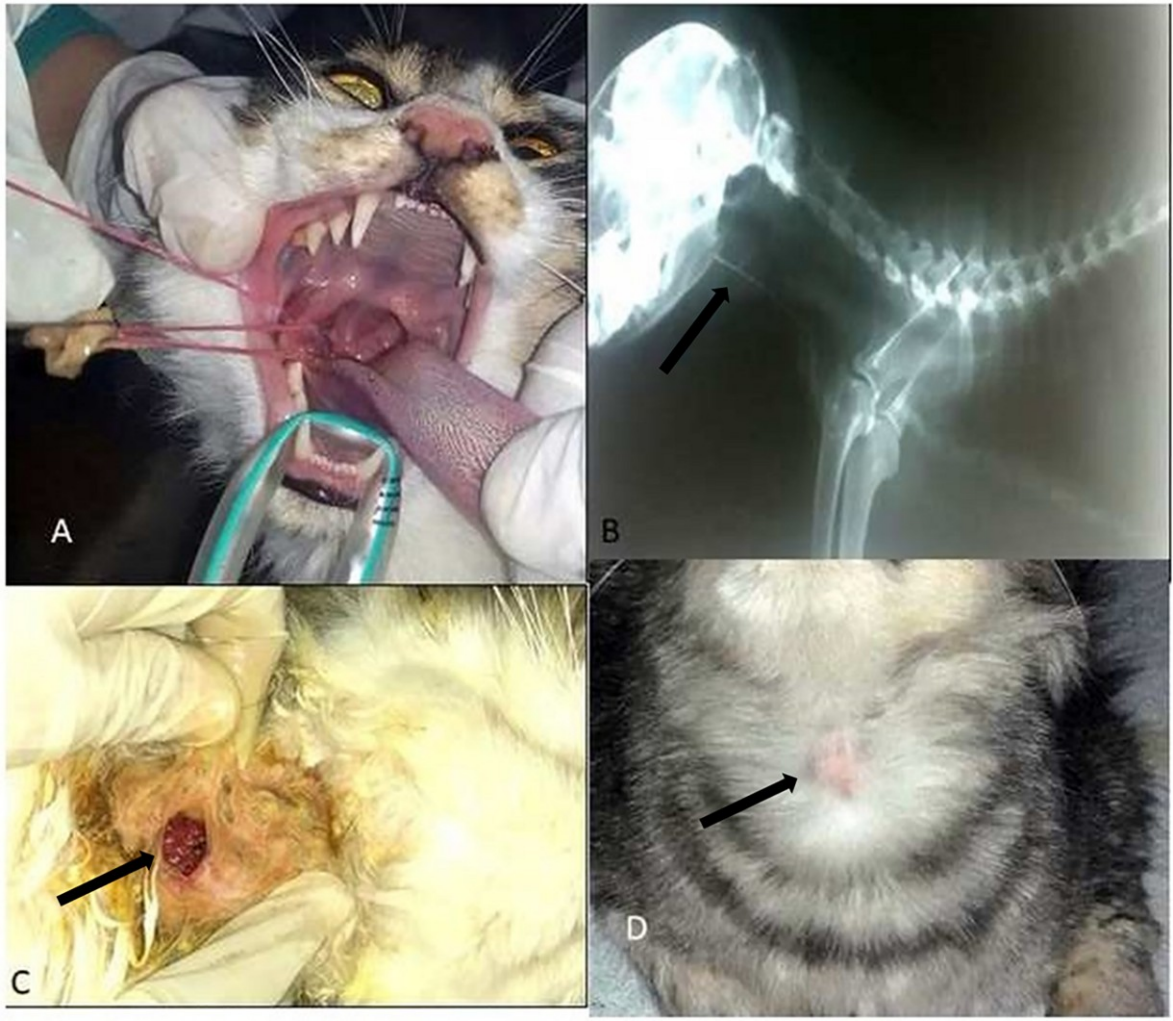
Complications of foreign bodies in the area of the oesophageal entrance (cervical region of the oesophagus caudal to pharynx). a. Photographic picture showing passing of needle and attached thread in epiglottis. b. Lateral radiographic view of cat showing radiopaque needle barrelled to oesophagus (black arrow). c. Photographic picture showing needle (black arrow) passing through oesophageal wall and forming abscess. d. Photographic picture showing small abscess at the level of the cervical oesophagus (black arrow).

The highest occurrence of lodgement was in the oesophageal entrance, i.e., in the cervical region of the oesophagus caudal to the pharynx (19/30, 63.3%). Radiographic imaging showed radiopaque needle dorsal to the cricoid cartilage in 17 cats; 2 remaining cats had a bone as the foreign body. The findings in these 19 cats were as follows: Thirteen cats presented with sewing needle protruding from the oral cavity, one cat had needle protrusion through the epiglottis, one cat had a needle passing through oesophageal wall, two cats had abscess formation, and two cats had bone foreign bodies. (Figs 1 and 2) (S6 and S7 Figs).

**Fig 3:**
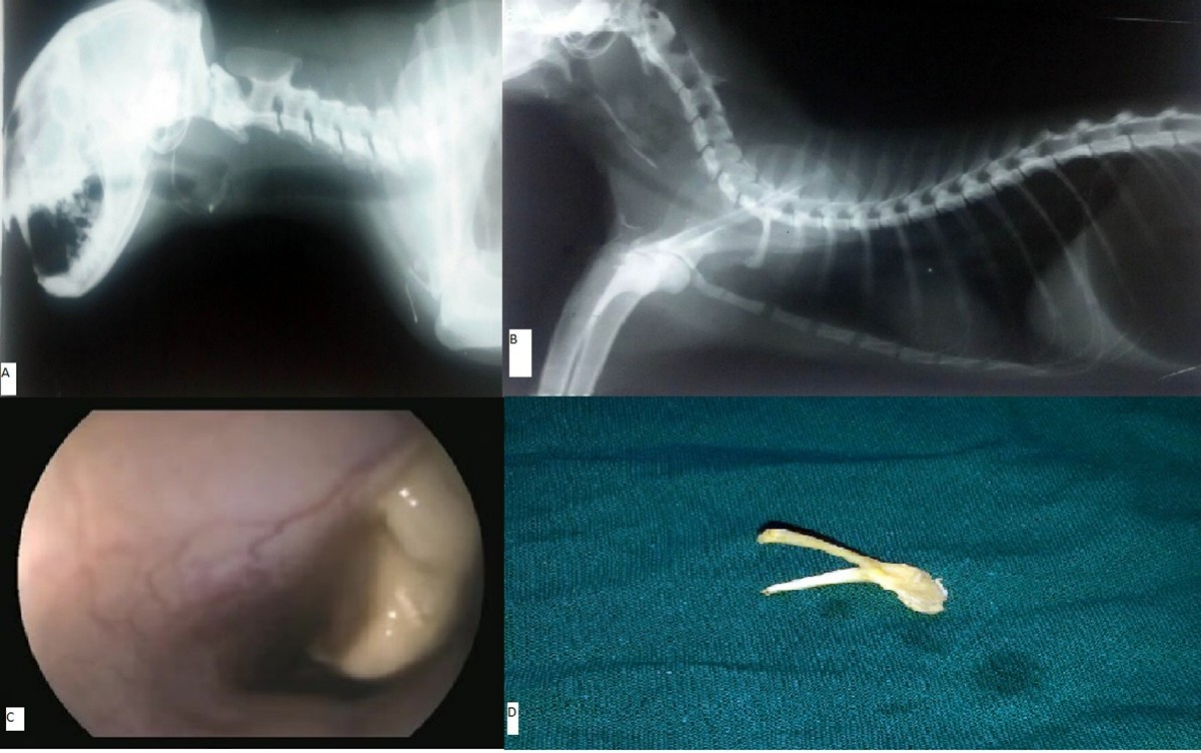
Radiographic and endoscopic views of foreign bodies in the mid-cervical oesophagus. a. Lateral radiographic view showing radiopaque bone structure in the cervical region of the oesophagus. b. Lateral radiographic view showing radiopaque bone fragments in the mid-cervical region of the oesophagus. c. Oesophagoscopy showing bone in the cervical region of the oesophagus. d. Bone after retrieval.

Foreign bodies in the mid-cervical region of the oesophagus were seen in 3/30 cases (10%). Bone was incriminated in all three cases, as shown in Fig 3. (S1, S2 and S4 Figs).

**Fig 4:**
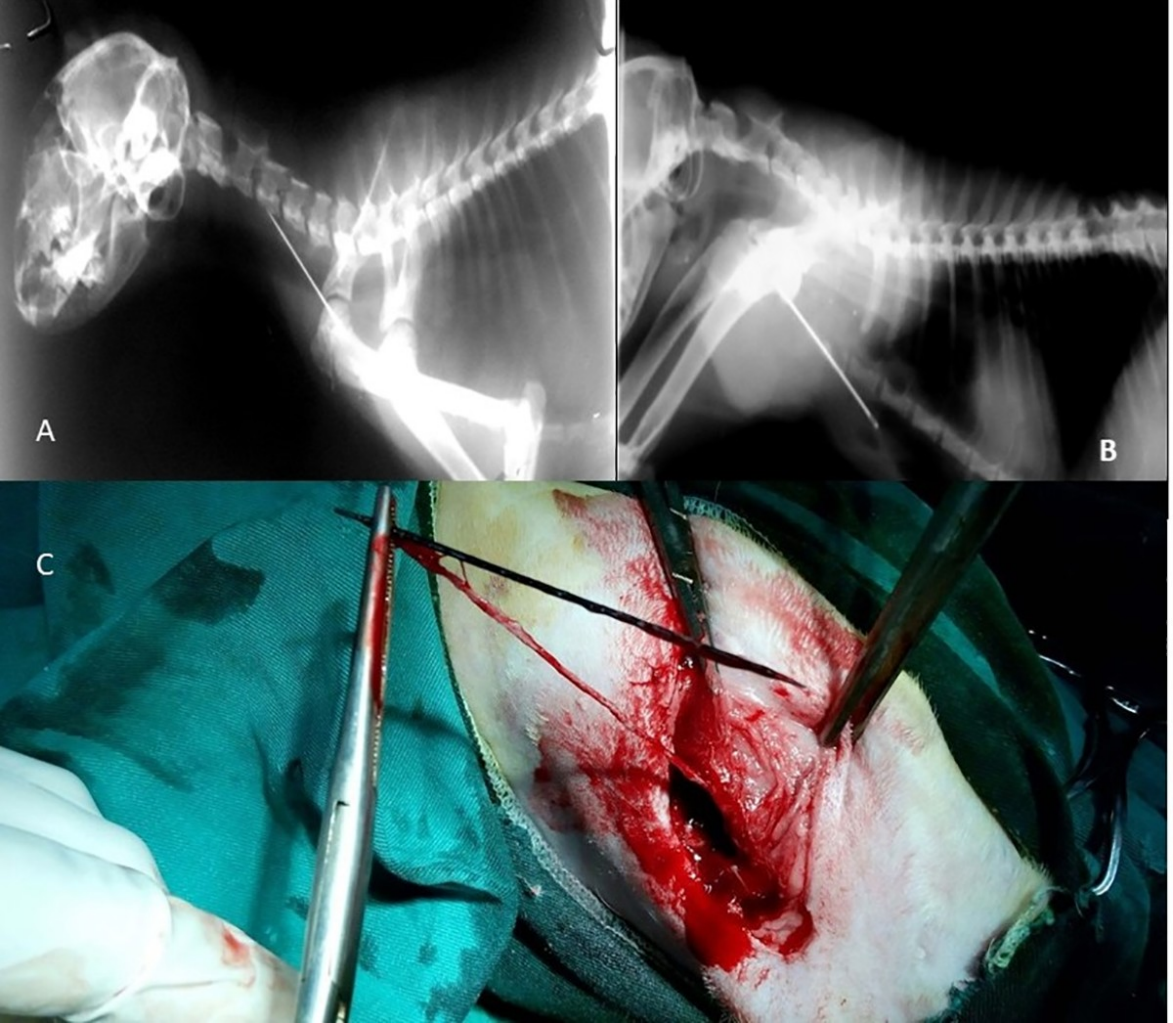
Foreign body lodgement at the thoracic inlet. a. Lateral radiographic view showing penetrating needle at the level of the thoracic inlet. b. Lateral radiographic view showing radiopaque needle with soft tissue abscessation from the axilla to the mammary glands. c. Removal of a needle with its suture material from the pectoral muscles.

Foreign bodies in the terminal region of the oesophagus, i.e., at the thoracic inlet, were observed in 8/30 cases (26.7%). Two of eight cats were admitted with the needle perforating at level of the 6th to 7th cervical vertebrae and forming a soft tissue reaction in the pectoral muscles (Fig 4) (S3 and S5 Figs)

### Therapeutic plan and complications

A treatment plan was assigned to each cat according to the lodgement site and nature of the foreign body as shown in Table 2. Removal of the foreign body was performed during endoscopic examination in 5/30 cases; removal was achieved using forceps in 17/30 cases, whereas full surgical intervention to remove the foreign body was performed in 8/30 cases. All cats made a full recovery.

Complications were identified as follow: one cat had needle protrusion through the epiglottis, one cat had a needle passing through oesophageal wall and two cats had bone foreign bodies. Two cats were admitted with the needle perforating at level of the 6th to 7th cervical vertebrae and forming a soft tissue reaction in the pectoral muscles (Figs 2 and 4). Overall complication rate was 6/30.

#### Establishment of the anatomical model

**Fig 5:**
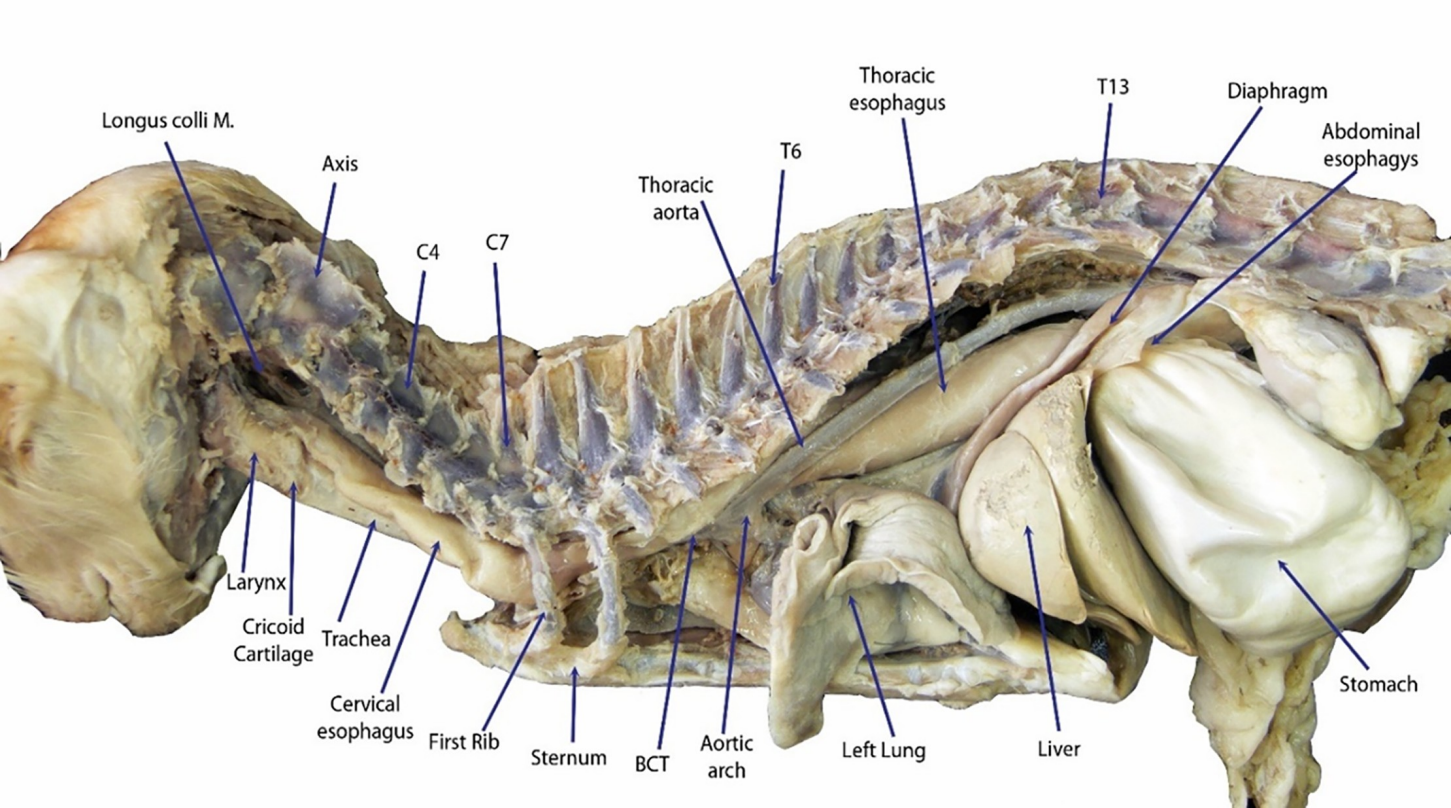
Dissected fixed cat, left side; the course of the entire oesophagus is shown.

**Fig 6:**
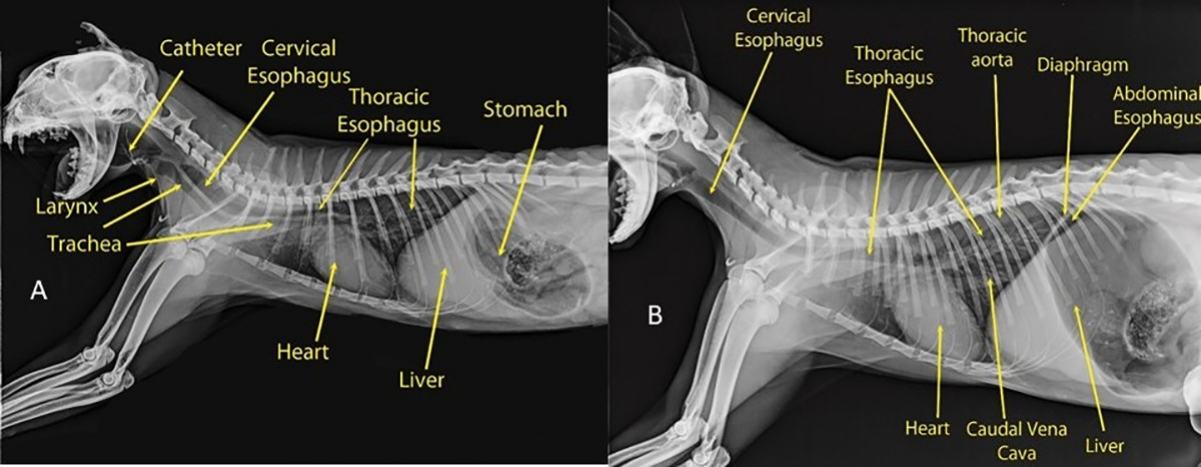
Negative contrast radiograph of a cat oesophagus. a) Left lateral view of a negative contrast radiograph of a cat oesophagus, showing its anatomy (visible Trachea). b) Left lateral view of a negative contrast radiograph of a cat oesophagus, showing its anatomy (visible Abdominal oesophagus and thoracic aorta).

**Fig 7:**
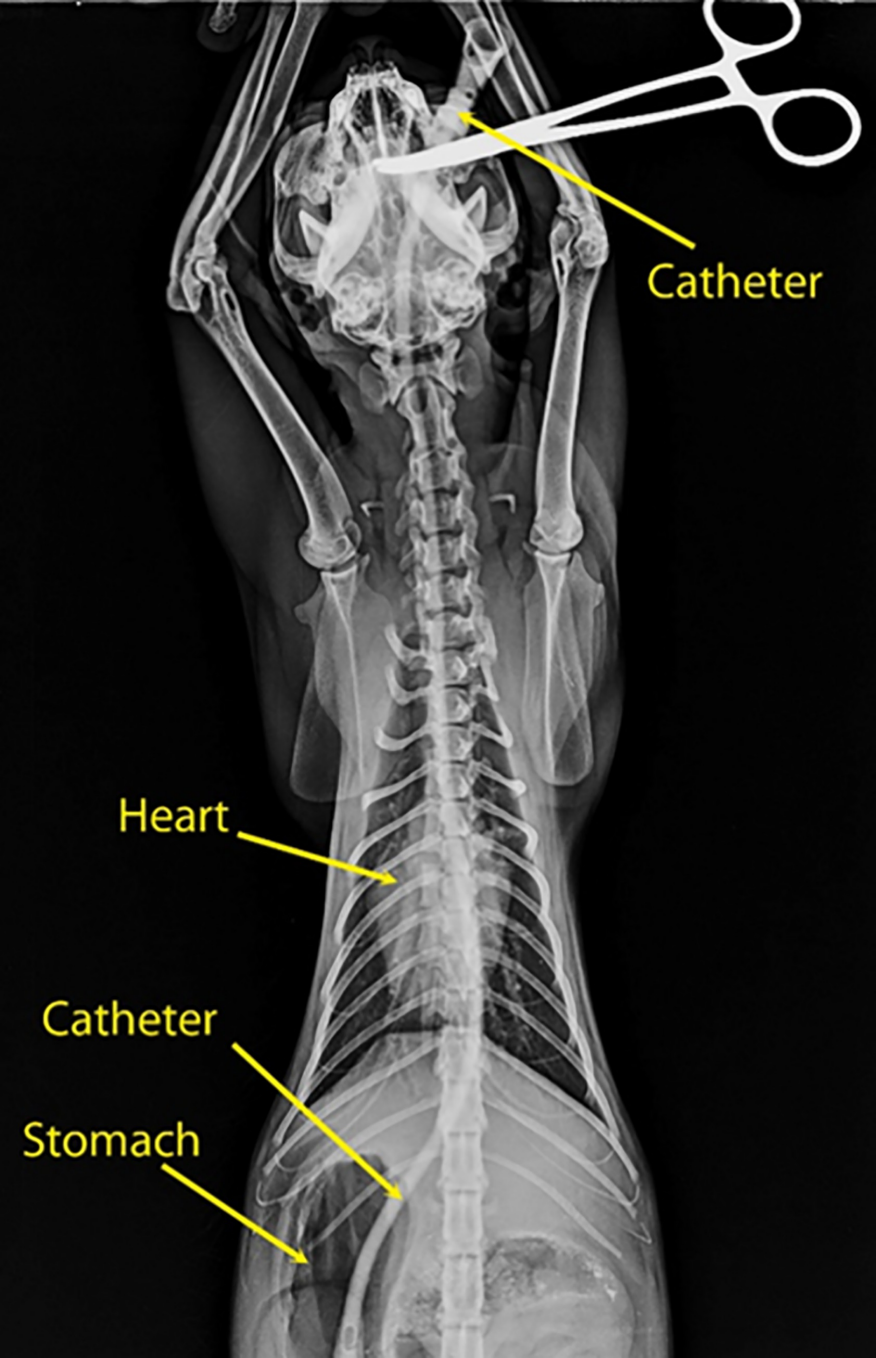
Ventro-dorsal view of a barium-filled catheter radiograph of a cat oesophagus, showing its anatomy.

**Fig 8:**
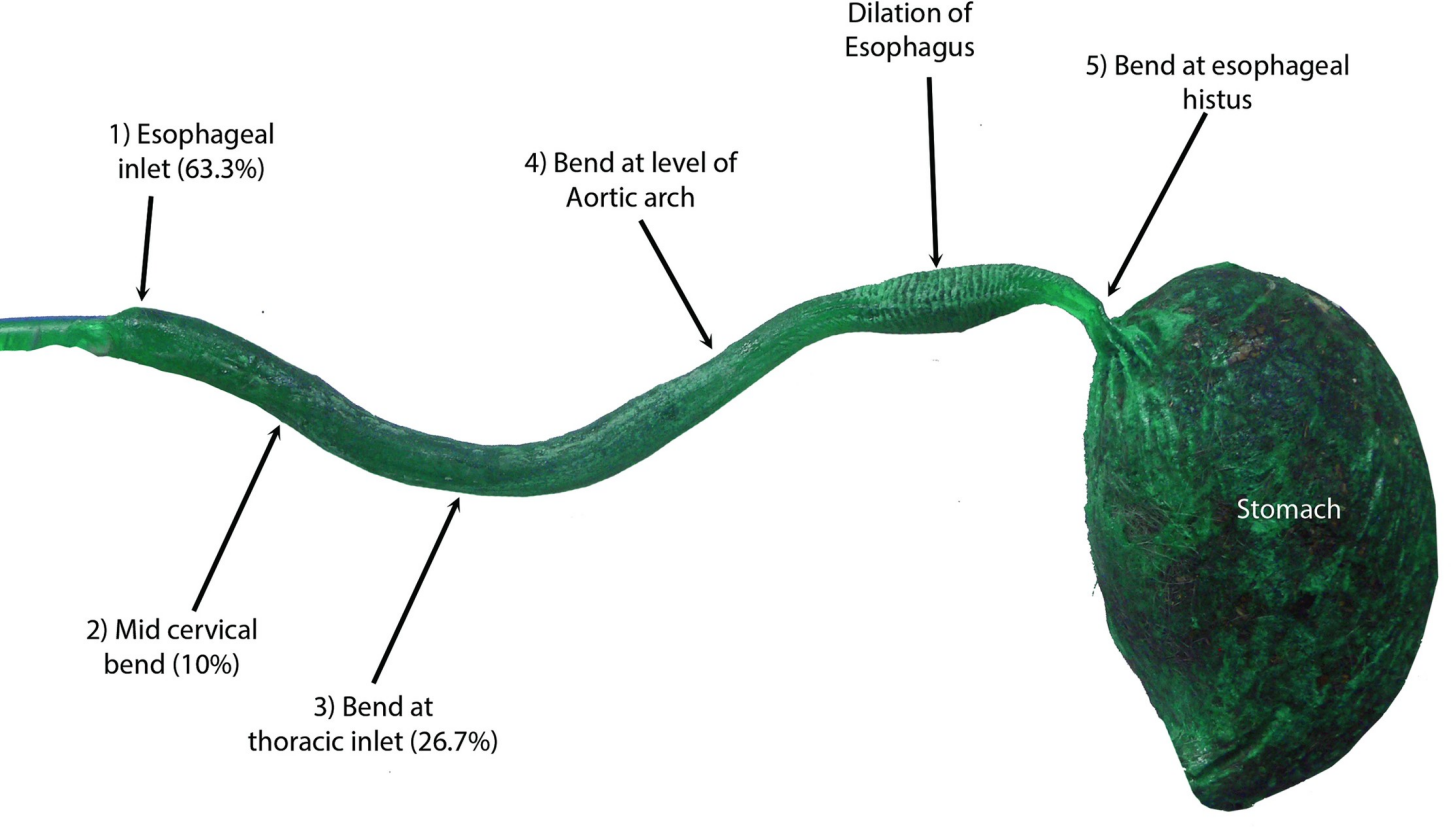
Kemapoxy 150 green-coloured corrosion cast of the oesophagus and stomach showing the main deviations of the oesophagus.

**Fig 9:**
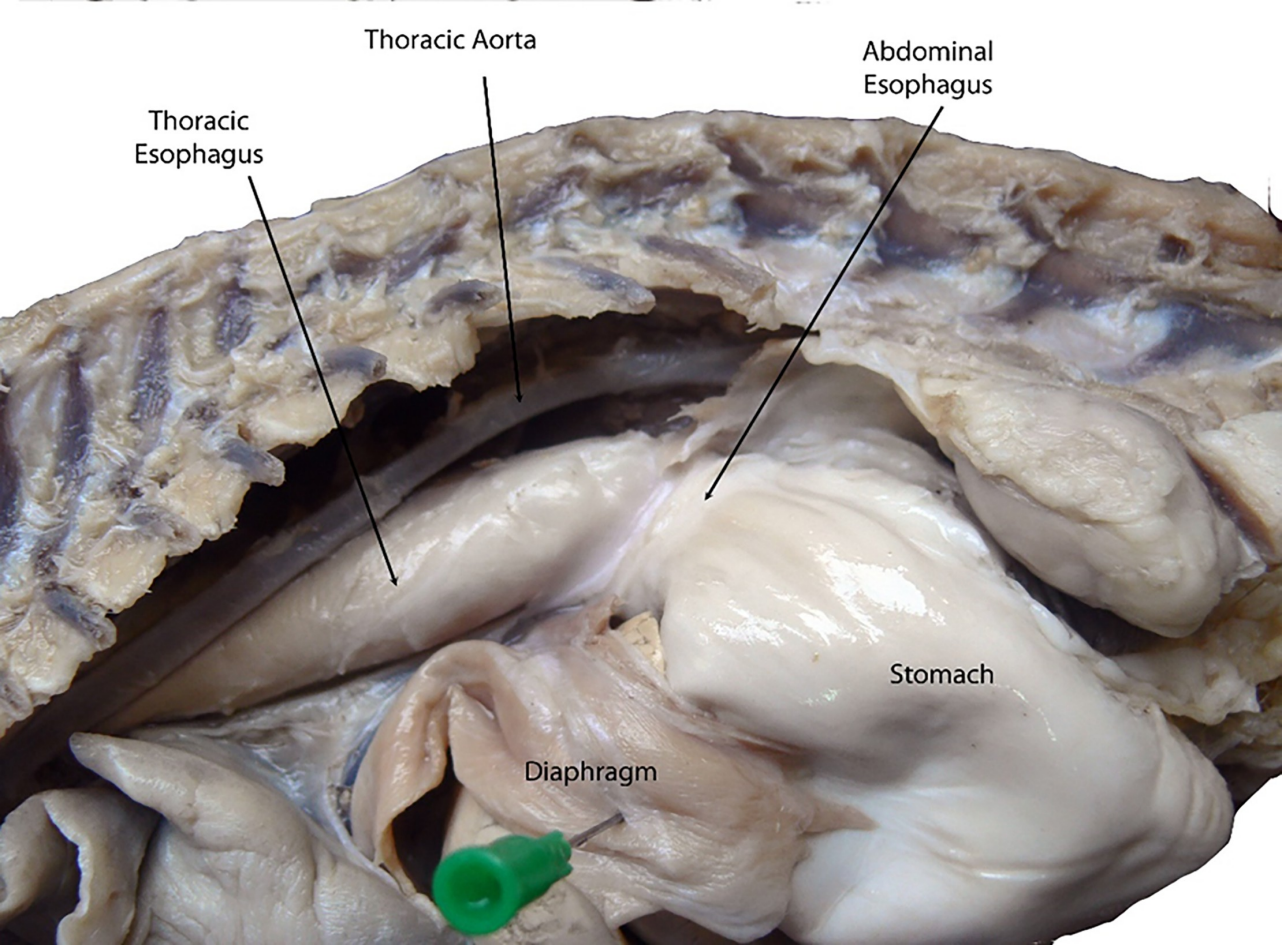
Dissected fixed cat, left side, with opened oesophageal hiatus in the diaphragm showing the terminal part of the oesophagus.

The oesophagus is composed of three segments: cervical, thoracic and abdominal. The cervical oesophagus originates at the caudo-ventral bending of the caudal end of the pharynx, just dorsal to the cricoid cartilage and ventral to its axis (Fig 5). The oesophagus continues dorsal to the trachea and ventral to the longus colli muscle until the level of the 4^th^ cervical vertebra, where it deviated towards the left side of the trachea. At the level of the 7^th^ cervical vertebra (Figs 5 and 6), the thoracic oesophagus bends caudo-dorsally to enter the thoracic cavity and continues in same direction, just dorsal to the brachiocephalic artery and brachiocephalic trunk and on the left side of trachea. At the level of the 6^th^ thoracic vertebra, the oesophagus inclines above the tracheal bifurcation and bends to the right of the ascending aorta, just above the aortic arch. The oesophagus continues in a dorso-caudal direction, with a slight tilting towards the left side (Fig 7), below the thoracic aorta in the mediastinum to penetrate the diaphragm through the oesophageal hiatus just below the 13^th^ thoracic vertebra. The thoracic oesophagus has a wide lumen in the area below the thoracic aorta, as shown in Fig 8. The abdominal oesophagus (Figs 5 and 9) is short and curves downward above the dorsal border of the liver to enter the stomach.

## Discussion

In the veterinary setting, cases of ingested foreign bodies, either complicated or uncomplicated, are common in cats; however, most are easily identified and treated successfully [3]. The risk factors for oesophageal foreign body's ingestion in cats include solitary play behaviour, *consumption of* non-food items, and consumption of chicken bones [7]. Furthermore, the anatomy of the cat oesophagus, which is similar to a curvilinear, narrow small tube [1], can result in lodgement when a sharp, hard, long object is encountered.

### Signs and physical findings

In the present study, signs and physical findings were similar to other reports [16]. These signs are widely accepted as associated with oesophageal foreign bodies in cats but are also observed in other conditions and diseases [4]. Male cats appeared to be approximately equally affected as female cats. Pratt et al., 2014 [16] reported similar findings, with a median age at lodgement of one and a half years. The witnessing of ingestion of the foreign body by the owner aids in making a presumptive diagnosis [5].

### Diagnostic aids

Foreign bodies identified in this study were radiopaque. Radiography is a valuable diagnostic tool for confirming the presence of a foreign body and its location [8]. Endoscopy has also gained wide popularity in such cases as it not only aids in diagnosis [5] but also can permit endoscopic extraction of object [10].

### Characterization of the nature and location of foreign bodies

Sewing needles and bones were the types of foreign body identified in this study. Generally, in feline practice, a wide variety of ingested items are observed; however, bones, needles and small toys are the most commonly ingested objects [17].

To better understand the locations of foreign body lodgement in the oesophagus and the oesophageal anatomical course, an anatomical model was created. The foreign bodies in the oesophagus were found in three main areas, namely, the oesophageal entrance caudal to the pharynx, the mid-cervical region of the oesophagus and the thoracic inlet. Our anatomic model showed four sites of inclinations, namely, at the oesophageal inlet caudal to the pharynx, at the thoracic inlet, at the level of aortic arch and at the oesophageal hiatus. Percentage of lodgement was higher in oesophageal inlet caudal to pharynx (63.3%) followed by thoracic inlet (26.7%). Based on this model, our clinical signs are predicted and consistent with anatomic feature. The oesophagus was divided into these three areas in the current work, similar to some other studies of domestic animals [1,2], including cats [18]. In other work, four narrow areas were reported as typically present in the oesophagus: directly caudal to the pharynx (superior oesophageal sphincter), at the cardiac base, at the thoracic inlet and the distal portion of oesophagus [19].

The highest occurrence among the lodgement sites was in the oesophageal entrance caudal to the pharynx (63.3%) followed by the thoracic inlet (26.7%). These findings are consistent with the above-described narrow and sharply-angled anatomy of the cat oesophagus. Pratt et al. 2014 [16] found that 29.7% of cat needle foreign bodies were in oropharynx and 51.4% were in the upper digestive tract and remaining 18.7% were in lower digestive tract. In our study, highest percentage was in oesophageal entrance caudal to the pharynx followed by the thoracic inlet and our anatomical model showed four area of inclination where if entrapment occurs, these areas may be expected to have highest percentage–they serve as natural road narrowing that when accidents happen, they slow down the traffic-. Early admission, nature of foreign body–bone- and natural inclinations of cats' oesophagus may play a role in hindering foreign body passage to lower digestive tract. Moreover, Pratt et al. 2014 [16] found that sewing needles were more common in the oropharynx than in the upper gastrointestinal tract, which they attributed to anatomical barriers and the nature of the cat gag reflex acting as physiological deterrent. However, the present study did not focus exclusively on sewing needles, as did another report [16], which may explain the different results. Furthermore, fishhooks were reported to lodge in the proximal oesophagus in the study by Binvel et al., 2018 [10], whereas another study reported the presence of foreign bodies found predominantly in the pharyngeal area, with only a small number of foreign bodies detected in the caudal oesophagus [20]. Presumably, the type, shape and sharpness of the foreign body factor into these different reports. After they are swallowed, sharp items can readily implant in the least expandable areas of the oesophagus [10].

### Establishment of the anatomical model

In this study, most of the foreign bodies lodged in the oesophageal entrance caudal to the pharynx, followed by the thoracic inlet. In the constructed anatomical model, four sites of inclination were detected, namely, at the oesophageal inlet caudal to the pharynx, at the thoracic inlet, at the level of aortic arch and at the oesophageal hiatus. These findings are in accordance with other studies [18, 21]. The anatomical findings of this study are consistent with the clinical findings, specifically, the sites of foreign body lodgement, as these regions are naturally inclined. Moreover, the sharp angles in the cat oesophagus might also play a role in the rate of complications.

### Treatment plans and complications

Oesophageal foreign bodies are often associated with complications, such as perforation of esophagus, stricture, inflammation and aspiration pneumonia [22]. The rate of complications in this study is 6/30 and in accordance with other reports [10]. Abscess formation and perforations are among the most common complications associated with oesophageal foreign bodies [6]. Two of eight cats were admitted with the needle perforating at level of the 6th to 7th cervical vertebrae and forming a soft tissue reaction in the pectoral muscles; our anatomic model shows the thoracic oesophagus bends caudo-dorsally to enter the thoracic cavity and continues in same direction. This might be attributed to angel at which foreign body punctured ventrally and direction of its sharp side as well as natural bending. No deaths were recorded among the studied animals; prognosis and mortality due to foreign bodies are dependent on the nature of the foreign body, the lodgement site and associated complications. For instance, perforation of the cervical region of the oesophagus was found to be associated with up to 6% mortality; this percentage increased when the foreign body was lodged caudally [23].

The selection of a therapeutic plan was performed in consideration of the location and type of the foreign body and the complications involved. Endoscopy to aid in removal of the foreign body was performed in five cats (16.6%) successfully. Endoscopic removal has been advocated in many studies as a treatment method, especially in cases where the foreign body is lodged in the pharyngeal area [20]. The reported success rates of this method are high, ranging from 65 to 95% [24]. Full surgical intervention was performed on eight cats (26%) due to the site of lodgement (at the thoracic inlet), the type of foreign body and the associated complications. The reported percentage of animals that require surgical intervention to remove foreign bodies is 15–38% [10, 25]. Removal of foreign body via forceps, a minimally invasive approach, was used in 17 cats (56%) in this study. Thirteen cats presented with a needle protruding through the oral cavity. Forceps-based retrieval has been described as a treatment method for foreign bodies in dogs, especially pharyngeal foreign bodies [26], and as common treatment for oral foreign bodies in dogs and cats [27].

## Conclusion

The results of this study suggest that the location of foreign bodies in the cat oesophagus is strongly related to the anatomic features of the oesophagus. Unlike the oesophagus of other domestic animals, the feline oesophagus has several sharp angles, which contribute to entrapment of foreign bodies. Radiographic imaging remains the most frequently used diagnostic modality to determine the lodgement site of radiopaque foreign body and its nature. The complication rate associated with sharp foreign bodies is higher for sewing needles than for bone.

## Supporting information

S1 Fig Sewing needle with attached thread after surgical removal. (JPG)

S2 Fig Oesophagoscopy showing bone in the esophagus. (JPG)

S3 Fig Outcropping of needle just after pectoral muscles incision. (JPG)

S4 Fig Lateral radiographic view showing radiopaque bone fragments in mid cervical part of esophagus. (JPG)

S5 Fig Ventrodorsal radiographic view showing radiopaque needle parallel to esophagus at thoracic inlet. (JPG)

S6 Fig Lateral radiographic view showing radiopaque needle at esophageal entrance caudal to pharynx. (JPG)

S7 Fig Endoscopic view showing bone fragment at esophageal entrance. (JPG)

S1 Data (PDF)

S2 Data (PDF)
