# Supplementary figures and images for: Oesophageal foreign bodies in cats: Clinical and anatomic findings
Source: PLoS One. 2020 Jun 2;15(6):e0233983. doi: 10.1371/journal.pone.0233983 (PMC7266337; doi:10.1371/journal.pone.0233983)

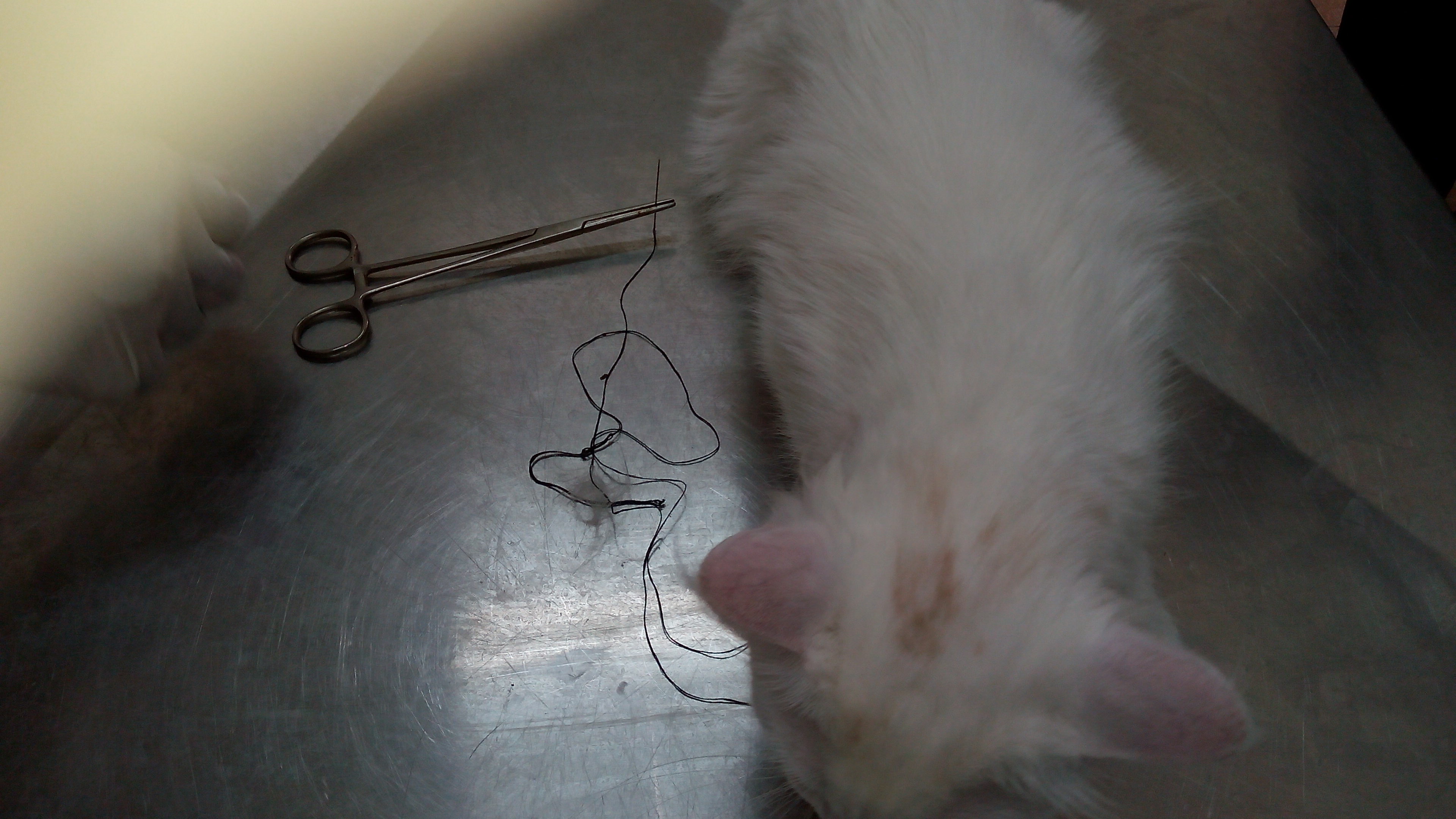

Supplement: S1 Fig — (JPG) [file pone.0233983.s001.jpg]

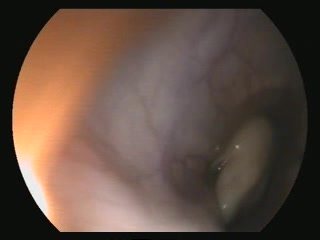

Supplement: S2 Fig — (JPG) [file pone.0233983.s002.jpg]

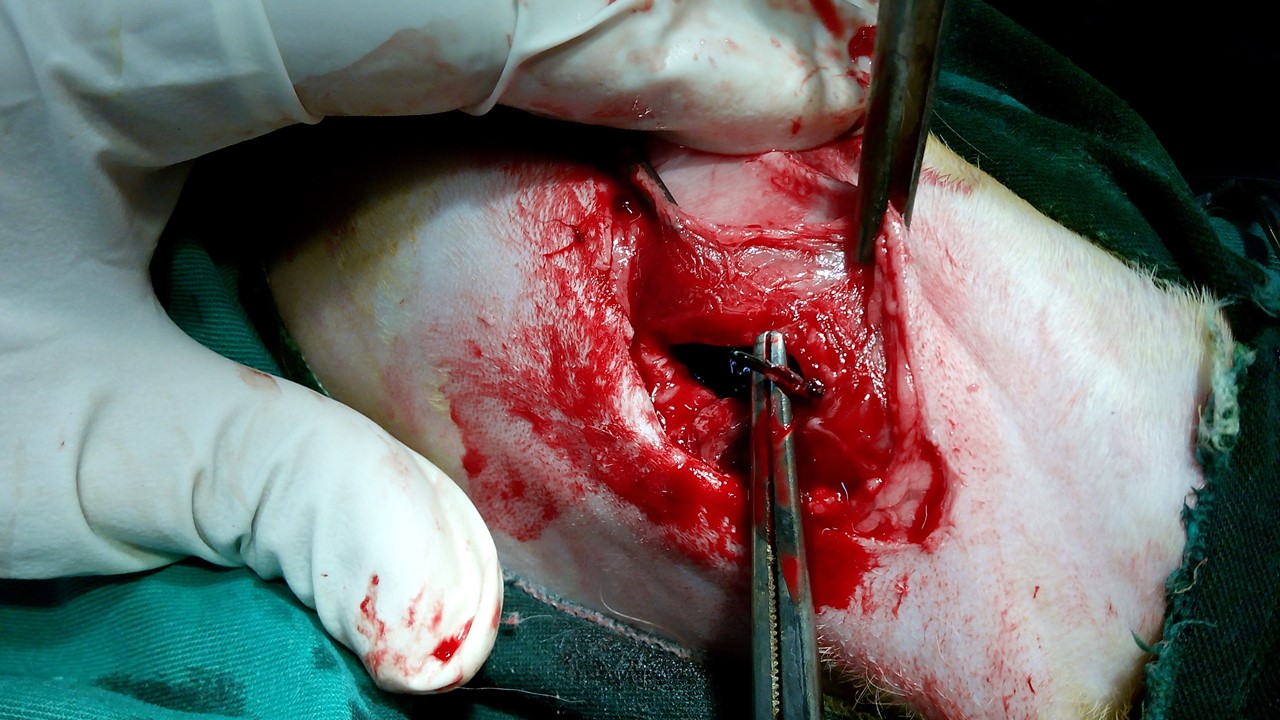

Supplement: S3 Fig — (JPG) [file pone.0233983.s003.jpg]

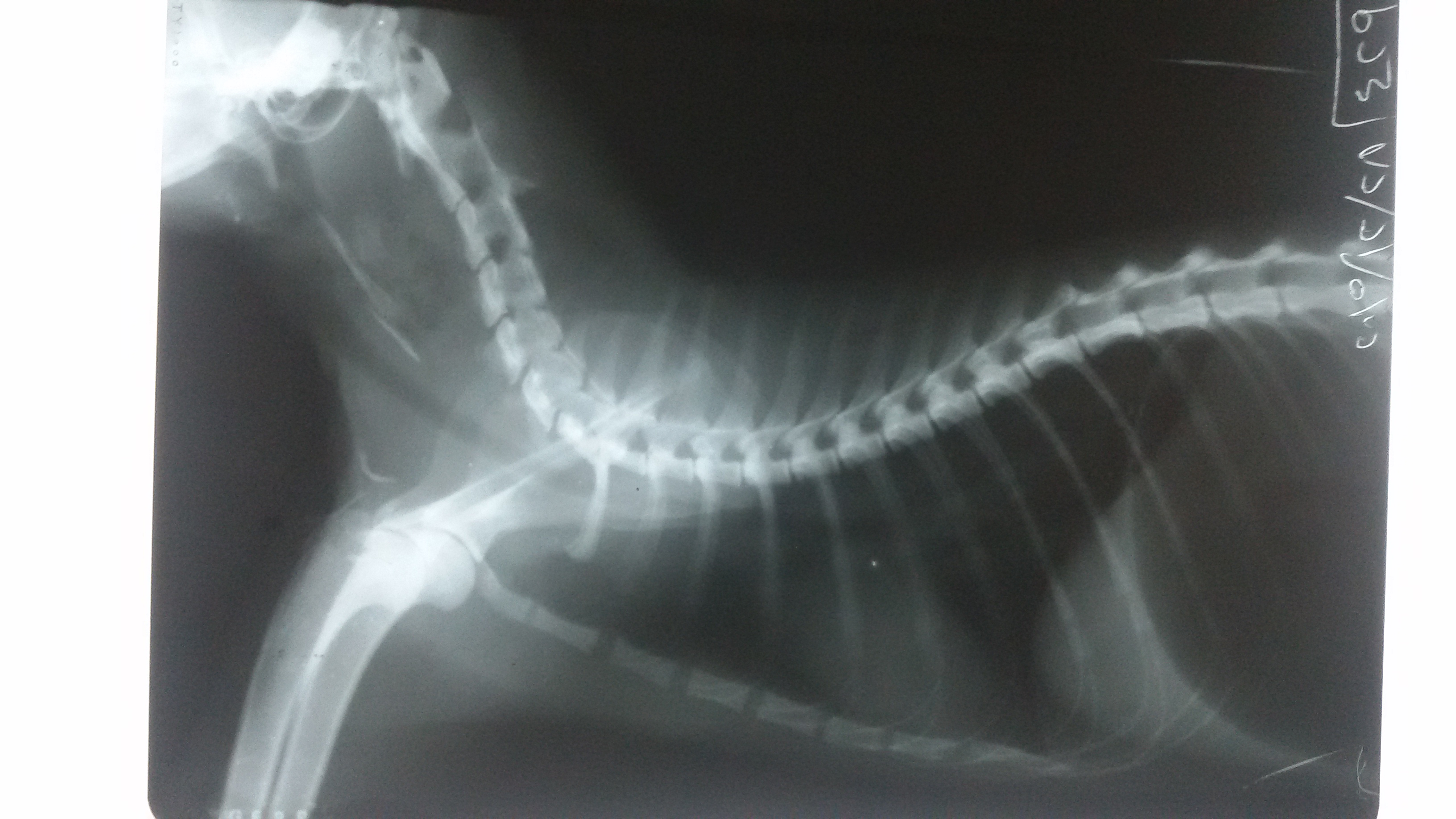

Supplement: S4 Fig — (JPG) [file pone.0233983.s004.jpg]

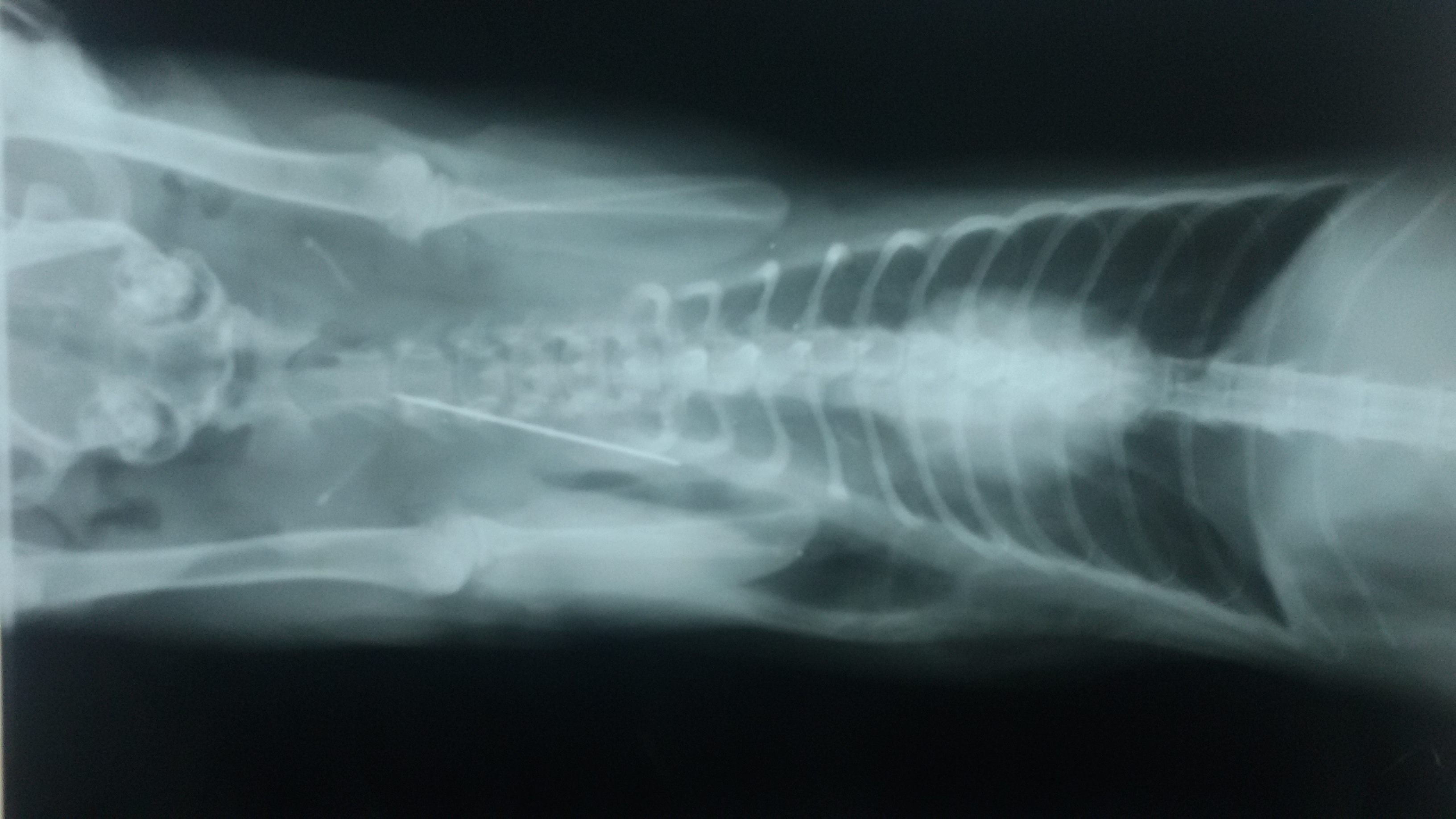

Supplement: S5 Fig — (JPG) [file pone.0233983.s005.jpg]

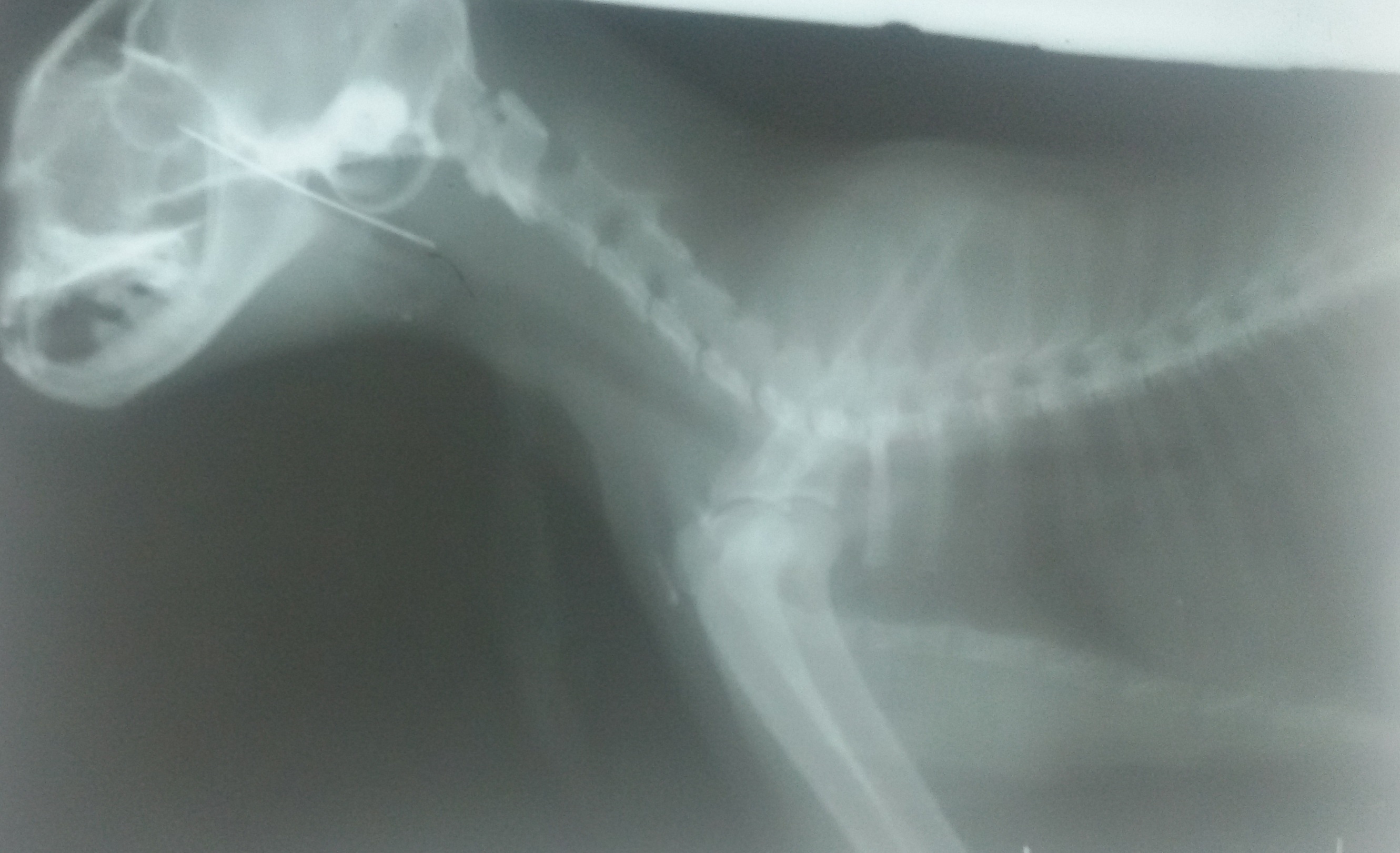

Supplement: S6 Fig — (JPG) [file pone.0233983.s006.jpg]

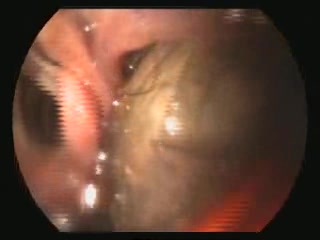

Supplement: S7 Fig — (JPG) [file pone.0233983.s007.jpg]
