# Supplementary material for: Oesophageal foreign bodies in cats: Clinical and anatomic findings
Source: PLoS One. 2020 Jun 2;15(6):e0233983. doi: 10.1371/journal.pone.0233983 (PMC7266337; doi:10.1371/journal.pone.0233983)
Supplement: S1 Data — (PDF) [file pone.0233983.s008.pdf]

November 20, 2018

Dear Nada Nabil,

Thank you for choosing Springer Nature Author Services. This manuscript, titled " Oesophageal foreign bodies in cats: clinical, radiologic, endoscopic and anatomic aspects," is very interesting. The paper was edited for grammar, phrasing, and punctuation. In addition, many edits were made to further improve the flow and readability of the text. Below, we highlight the areas of this paper that we focused on in our edit.

Efforts were made to ensure the appropriate level of formality and professionalism according to the tone expected in much academic writing.

Certain edits were made to remove redundant, repetitive or unnecessary phrasing and to present the information in a more straightforward manner.

Some sentences were restructured to address overly complex structure or to revise potentially unclear phrasing.

Comments were left if further clarification would be helpful or confirmation of the meaning of the text was necessary. Please review these comments and all our changes carefully for more detailed suggestions, as well as to ensure that the final version of the manuscript is fully accurate.

Thank you again for using our editing services; we wish you the best of luck with your submission.

Best regards,

Kari P.
Senior Editor
Springer Nature Author Services
